# The skinny on cocaine: Insights into eating behavior and body weight in cocaine-dependent men^☆☆^^☆☆^

**DOI:** 10.1016/j.appet.2013.07.011

**Published:** 2013-12-01

**Authors:** Karen D. Ersche, Jan Stochl, Jeremy M. Woodward, Paul C. Fletcher

**Affiliations:** a University of Cambridge, Department of Psychiatry, Cambridge CB2 0SZ, UK; b University of Cambridge, Behavioural and Clinical Neuroscience Institute, Cambridge CB2 0SZ, UK; c Cambridge University Hospitals NHS Foundation Trust, Department of Gastroenterology, Addenbrooke’s Hospital, Cambridge CB2 0QQ, UK

**Keywords:** Dietary food intake, Body weight, Fat regulation, Cocaine dependence, Anthropometry, Impulsivity–compulsivity

## Abstract

There is a general assumption that weight loss associated with cocaine use reflects its appetite suppressing properties. We sought to determine whether this was justified by characterizing, in detail, alterations in dietary food intake and body composition in actively using cocaine-dependent individuals. We conducted a cross-sectional case-control comparison of 65 male volunteers from the local community, half of whom satisfied the DSM-IV-TR criteria for cocaine dependence (*n* = 35) while the other half had no personal or family history of a psychiatric disorder, including substance abuse (*n* = 30). Assessments were made of eating behavior and dietary food intake, estimation of body composition, and measurement of plasma leptin. Although cocaine users reported significantly higher levels of dietary fat and carbohydrates as well as patterns of uncontrolled eating, their fat mass was significantly reduced compared with their non-drug using peers. Levels of leptin were associated with fat mass, and with the duration of stimulant use. Tobacco smoking status or concomitant use of medication did not affect the significance of the results. Weight changes in cocaine users reflect fundamental perturbations in fat regulation. These are likely to be overlooked in clinical practice but may produce significant health problems when cocaine use is discontinued during recovery.

## Highlights

• Low body weight in cocaine users may not be due to the appetite suppressing effects of the drug.
• Chronic cocaine users report higher levels of uncontrolled food intake than healthy volunteers.
• Imbalance in fat intake and storage may explain the excessive weight gain during abstinence.

## Introduction

There is a wide belief that cocaine use suppresses appetite, thereby reducing body weight (Cochrane, Malcolm, & Brewerton, 1998). This view is supported by observations that problematic weight gain may rapidly emerge on cessation of regular cocaine use (Cowan & Devine, 2008), a distressing phenomenon that can lead to relapse. Many clinical interventions are therefore shaped by the belief that eating habits and weight-related problems are not an issue during cocaine use (VanBuskirk & Potenza, 2010), but rather occur in abstinent users to restore the weight that they previously lost due to a cocaine-induced suppression of appetite (Vanbuskirk & Potenza, 2010). As a consequence, community treatment services try to address drug users’ weight problems with educational interventions promoting healthy eating (Cowan & Devine, 2012). However, we argue that a more nuanced view is needed, one that acknowledges a major disturbance in eating behaviors and metabolism accompanying cocaine use.

Research in experimental animals indicates that cocaine’s anorexic effects are relatively transient (Balopole, Hansult, & Dorph, 1979), with intake of food delayed but not actually reduced (Cooper & Vanderhoek, 1993), and followed by a compensatory increase in the consumption of fat and carbohydrates (Bane, Mccoy, Stump, & Avery, 1993). Paradoxically, the weight gain generally associated with increased caloric density and fat intake is, however, not seen in cocaine-treated animals (Bane et al., 1993). Similar observations have also been noted in humans: regular cocaine users report eating fewer balanced meals than non-using peers, with an expressed preference for fatty foods, but no corresponding weight gain (Castro, Newcomb, & Cadish, 1987). Given that the significant weight gain following cocaine abstinence is not only a source of major personal suffering but also has profound implications for health and recovery, we suggest that there is a pressing need for a more detailed understanding of the effects of cocaine on dietary intake and body composition. This is an important consideration given that by far the most substantial health burden arising from drug addiction lies not in the direct effects of drug intoxication but in the secondary effects on physical health.

In the current study, we characterized key patterns of eating behavior and weight change in cocaine dependence. We measured circulating levels of leptin, body composition and self-reported eating habits in a sample of cocaine-dependent men and compared them to matched healthy non-drug using male volunteers. We hypothesized that chronic cocaine use is associated with changes in eating patterns, specifically with regard to the consumption of fat and carbohydrates that had been observed in cocaine-treated animals. We predicted that changes in dietary food intake are reflected in alterations of body composition.

## Methods

### Study sample

Sixty-five male volunteers participated in this study. They were recruited within the local community either upon referral from health care professionals, probation officers, advertisements or by word-of-mouth. Drug-using volunteers had to meet the DSM-IV-TR criteria for cocaine dependence (American Psychiatric Association, 2000) whereas control volunteers had to have no personal or family history of substance misuse disorders. Exclusion criteria for all volunteers included a lifetime history of a psychotic disorder; a neurological illness or a traumatic head injury; an autoimmune or a metabolic disorder; and a current infection with HIV. All volunteers consented in writing and were screened for current psychiatric disorders using the Mini-International Neuropsychiatric Inventory (Sheehan et al., 1998). Current and past psychopathology in the drug users was further evaluated using the structured clinical interview for DSM-IV (First, Spitzer, Gibbon, & Williams, 2002). Verbal intelligence was estimated in all volunteers using the National Adult Reading Test (Nelson, 1982). All participants further completed the Barratt Impulsiveness Scale (BIS-11, Patton, Stanford, & Barratt, 1995) and the Obsessive–Compulsive Inventory (OCI-R, Foa et al., 2002) to assess both impulsive and compulsive personality traits. The protocol was approved by the National Research Ethics Committee (NREC10/H0306/69, PI: KD Ersche).

All drug users satisfied the DSM-IV-TR (American Psychiatric Association, 2000) criteria for cocaine dependence. They were non-treatment seeking and actively using cocaine either in powdered (40%) or in freebase form (60%). They had been using cocaine for an average of 15.3 years (±9.0 SD), starting at the age of 19.2 years (±5.5 SD). On the testing day, urine samples tested positive for stimulants in all except four users, indicating that they consumed either cocaine or amphetamines within the detection window of 72 h (Preston et al., 2002). On the Obsessive-Compulsive Drug Use Scale (OCDUS, Franken, Hendriks, & Van Den Brink, 2002) they indicated moderate levels of cocaine-related compulsivity (mean score = 23.8 ± 10.7 SD). The majority of the drug user sample also met criteria for dependence on another substance (91% nicotine, 43% opiates, 29% alcohol, 20% cannabis, 3% amphetamines) and used other drugs sporadically (68% cannabis, 20% sedatives, 15% opiates, 14% ecstasy, 3% hallucinogens). Participants with co-morbid opiate dependence were either prescribed methadone (31%, mean dose: 55 mg ± 16.2SD) or buprenorphine (9%, mean dose: 3 mg ± 2.6SD), or were using street heroin on a daily basis (3%). A quarter of cocaine users reported taking prescribed medication, including narcotic-like pain relief (11%), antidepressants (9%), benzodiazepines (9%), and d-amphetamine (3%).

The healthy volunteers were screened for drug and alcohol abuse, but none met criteria for abuse or dependence. Thirteen percent were current tobacco smokers and 57% reported past tobacco smoking habits. Half of the healthy volunteer group (53%) reported having had social experiences with cannabis but never had used the drug regularly; none of them reported taking prescribed or illicit drugs on a regular basis and urine sampled on the testing day was negative for illicit substances.

### Procedures

Participants were examined in the Wellcome Trust Clinical Research Facility at Addenbrooke’s Hospital, Cambridge, U.K. A trained research assistant assessed participants’ eating behavior and habitual diets using validated instruments. The Food Frequency Questionnaire (FFQ) determines individuals’ habitual dietary food intake by measuring the frequency with which food items have been consumed over the past year (Bingham et al., 2001). The FFQ was initially developed for the European Prospective Investigation to evaluate the role of diet and nutritional status in cancer rates and has been widely used ever since (http://www.srl.cam.ac.uk/epic/nutmethod/FFQ.shtml). It contains a list of 131 food items that are commonly consumed in the U.K. as well as specific questions regarding the fat content of dietary products and the types of fat used for cooking. For each food item, the average consumption per day was estimated on the basis of the reported portion size and the frequency that each food item was consumed using specifically-developed software programs (Welch, Luben, Khaw, & Bingham, 2005; Welch et al., 2001).

The Three-Factor Eating Questionnaire (TFEQ), originally developed by Stunkard and Messick (1985) and revised by Karlsson, Persson, Sjostrom, and Sullivan (2000), consists of 18 items to measure three different aspects of eating behavior: *restrained eating* (deliberate restriction of food intake to control body weight), *uncontrolled eating* (tendency to eat more than intended by losing control over food intake), and *emotional eating* (tendency to eat in response to emotional cues). Participants indicate the extent to which these behaviors reflect their eating pattern on a 4-point scale.

Anthropometric measurements were acquired by a metabolic physiologist using both manual measurements and dual-energy X-ray absorptiometry scans (DXA; GE Lunar Prodigy, Madison, WI) to determine fat mass, non-bone lean mass and bone mineral density. Levels of plasma leptin were measured in light of the hypothesized influence on body composition using an RIA kit (Antibodies & Standards from R&D Systems, Abingdon UK) with an in-house two-site microtitre plate-based DELFIA assay with a between batch imprecision of 7.1% at 2.7 ng/ml, 3.9% at 14.9 ng/ml, 5.7% at 54.9 ng/ml (in-house data).

### Statistical analysis

Responses on the FFQ were processed using a modified version of the DINER and CAFE programs (Welch et al., 2001, 2005). All data were analyzed using the Statistical Package for Social Sciences (SPSS.V20; IBM). Group difference in demographics were analyzed using *t*-tests, except for leptin levels, which did not meet parametric requirements, so the Mann–Whitney *U*-test was used. As in 6% of cases anthropometry measures were taken by a different member of staff, analysis of co-variance was used for group comparisons in anthropometry and the categorical variable ‘staff’ was included as a covariate. Questionnaire data were analyzed using multivariate analysis of variance. To statistically control for significant group differences in energy and alcohol intake in the FFQ analysis, we included energy (kcal) and alcohol (g) as covariates in the model, and we also corrected the significance levels for multiple testing at group comparison level using the Bonferroni method. We conducted subsequent analysis to verify that results were not confounded by smoking status (never, present, past) or concomitant medication by including both variables as additional covariates in the model. We also performed sub-group analysis between cocaine-dependent men with and without opiate dependence or with and without alcohol dependence to examine potential effects of co-morbidity. We used multiple regression analysis to further examine the extent to which dietary fat accounted for participants’ high calorie intake. If not stated otherwise, Pearson’s correlation co-efficient was calculated for descriptive purposes. All statistical tests were two-tailed and a significance level of 0.05 was assumed. As the personality measures were included after the start of the study, BIS-11 data was not available from nine volunteers (4 controls, 5 cocaine) and OCI-R was not available from 5 volunteers (1 control and 4 cocaine).

## Results

### Demographics and clinical characteristics

The two groups only included male participants who were age-matched (*t*_63_ = 0.5, *p *= 0.596). As commonly seen in drug-dependent populations, the cocaine group had a lower verbal IQ (*t*_59_ = 5.6, *p *< 0.001) and spent less time in education (*t*_63_ = 3.4, *p *< 0.001) compared with their healthy peers. In keeping with the notion that impulsivity–compulsivity traits are associated with addiction vulnerability (Dalley, Everitt, & Robbins, 2011; Ersche et al., 2013), the cocaine-dependent individuals reported significantly higher levels of trait impulsivity (*t*_54_ = −6.8, *p *< 0.001) and compulsivity (*t*_58_ = −2.6, *p *= 0.012) compared with their healthy peers. All drug-dependent participants were asked about their motivation for using cocaine; the most common reasons were ‘liking the effects’ (55%), ‘don’t know’ (42%), ‘distracting myself’ (15%), ‘need it every day to get me going’ (12%), ‘boredom’ (12%), ‘socializing with others’ (12%), and ‘sexual activity’ (9%). None of the users mentioned ‘losing weight’ or ‘suppressing appetite’ as a reason for using cocaine, which concurs with previous studies indicating that relationships between stimulant use and body dissatisfaction, dieting behavior or attempts to lose weight were gender-specific and not observed in male users (Cochrane et al., 1998; Parkes, Saewyc, Cox, & Mackay, 2008). Vital signs such as blood pressure and pulse did not differ between the two groups, suggesting that the cocaine-dependent participants were not intoxicated during the study. Mean levels of leptin were lower in the cocaine group (see Table 1), albeit not significantly.

### Anthropometry

Conventional measures of anthropometry such as the body mass index (BMI), waist–hip ratio, or skinfold thickness did not identify differences between the groups (see Table 1). The DXA scan, however, revealed altered body composition in the cocaine group, as reflected by a significantly lower ratio of fat mass to fat-free mass in cocaine-dependent men (*t*_63_ = 2.3, *p *= 0.026). This ratio remained significant when tobacco smoking and concomitant medication status were included as covariates (*F*_1,61_ = 6.3, *p *= 0.015). Analogous to the BMI, we calculated indices for fat mass (FMI), fat-free mass, and lean mass for each participant by dividing each measure by individuals’ heights squared. Group comparisons confirmed that the change was specific for body fat (*t*_63_ = 2.2, *p *= 0.031) and not seen for indices of fat-free mass (*t*_63_ = 0.2, *p *= 0.853) or lean mass (*t*_63_ = 0.1, *p *= 0.918). Again, the reduction in body fat in the cocaine group remained significant when tobacco smoking and medication status were statically controlled for (*F*_1,61_ = 6.4, *p *= 0.014). Subgroup comparisons between cocaine-dependent men with and without opiate dependence or with and without alcohol dependence did not reveal any significant differences on any of the aforementioned measures (all *p *> 0.1). Both BMI and FMI were significantly correlated with levels of leptin in cocaine-dependent individuals (Spearman’s *r*_BMI_ = 0.83 and *r*_FMI_ = 0.91, both *p *< 0.001) and in control volunteers (Spearman’s *r*_BMI_ = 0.72 and *r*_FMI_ = 0.83, both *p *< 0.001). In the cocaine group, leptin levels were also correlated with the duration of stimulant use (Spearman’s *r *= 0.39; *p *< 0.05); albeit the duration of stimulant use was not correlated with BMI or FMI.

### Eating patterns

Habits of skipping breakfast were more frequently reported in cocaine-dependent men (86%) compared with healthy men (20%) (*χ*^2^ = 28.2, *p *< 0.001). The cocaine users also reported a higher intake of alcohol (*t*_42.5_ = −2.5, *p *= 0.017) and caloric intake (*t*_61.1_ = 6.9, *p *= 0.033), as measured by the FFQ, but which were statistically controlled for in the analysis of dietary data. Overall, cocaine users’ diets differed significantly from those of their non-drug using peers (*F*_56,6_ = 11.2, *p *= 0.003; Wilks’ Lambda = 0.003). Specifically, cocaine users consumed significantly more fatty foods (*F*_1,61_ = 19.8, *p *< 0.05), as reflected by increased levels of both monounsaturated (*F*_1,61_ = 15.9, *p *< 0.05) and saturated fatty acids (*F*_1,61_ = 22.6, *p *< 0.05). The cocaine group also reported consuming significantly more carbohydrates compared with the control group (*F*_1,61_ = 15.7, *p *< 0.05), but less sugar such as fructose (*F*_1,61_ = 16.8, *p *< 0.05) and glucose (*F*_1,61_ = 16.9, *p *< 0.05). Subsequent analysis with tobacco smoking status and prescribed medication both included as additional covariates did not change the results. It is noteworthy that the reported dietary intake did not differ between cocaine-dependent men with and without opiate dependence or with and without alcohol dependence, respectively (all *p *> 0.1).

Multiple regression analysis showed that the fat content of the diet explained 91% of the variance of the overall energy intake in the cocaine group (*R*^2^ = 0.907, *F*_1,33_ = 332.85, *p *< 0.001), but not in the control group. Carbohydrates explained 94% of the consumed dietary calories in the diets of the healthy volunteers (*R*^2^ = 0.944, *F*_1,28_ = 485.94, *p *< 0.001).

Besides the differences in dietary intake, cocaine-dependent men also reported differences in the pattern of food consumption compared with the control volunteers, as measured by the TFEQ. Cocaine-dependent men reported exerting less control over food intake (*F*_1,63_ = 11.6, *p* = 0.001) and less dietary restraint to influence their body weight (*F*_1,63_ = 6.9, *p* = 0.011) compared with their healthy peers. The inclusion of tobacco smoking and prescribed medication did not change the significance on both measures. There was also no evidence that co-morbid dependence on opiates and/or alcohol affected the results.

### Explorative relationships between personality traits and eating patterns

**Fig. 1:**
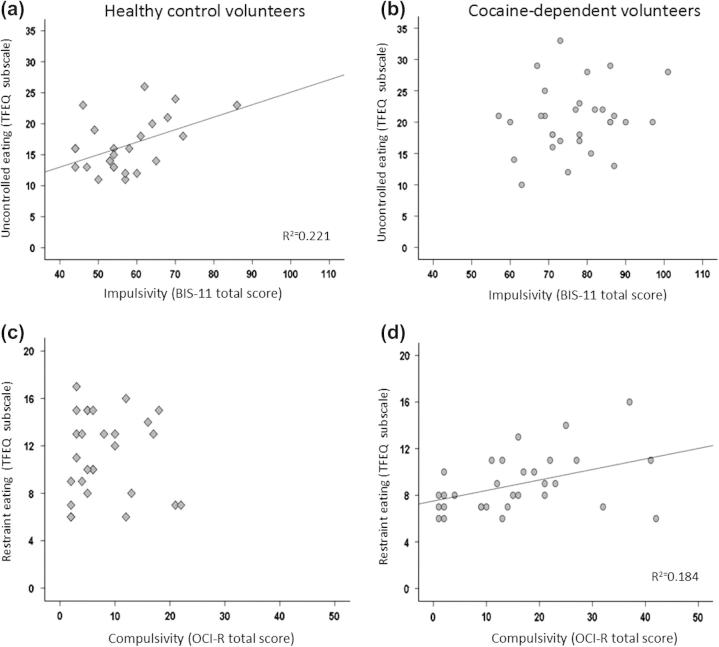
Correlations between eating behavior, as measured by the Three-Factor Eating Questionnaire (TFEQ) and personality traits of impulsivity and compulsivity, as measured by the Barratt Impulsiveness Scale (BIS-11) and the Obsessive–Compulsive Inventory (OCI-R). (a and b) impulsive personality traits are significantly associated with uncontrolled eating in healthy men, but not in cocaine-dependent men. (c and d) compulsive personality traits are associated with deliberate restriction of food intake to influence body weight in cocaine-dependent men but this relationship is not seen in healthy men.

As shown in Fig. 1, impulsive and compulsive personality traits were differentially associated with eating behavior in the two groups. Consistent with the literature, impulsive personality traits were significantly associated with uncontrolled eating in healthy volunteers (*r* = 0.47, *p* < 0.001) (Yeomans, Leitch, & Mobini, 2008), but no such relationship was seen in the cocaine group (*r *= 0.20,*p *= 0.282). Uncontrolled eating in the control group was further associated with individuals’ dietary fat intake (*r *= 0.34, *p *= 0.006), but this relationship was only marginally significant in the cocaine group (*r *= 0.30, *p *= 0.078). By contrast, restrained eating to influence body weight was significantly associated with compulsive personality traits in the cocaine group (*r *= 0.43, *p *= 0.016); but compulsivity was not related to fat consumption in either group (cocaine: *r *= −0.02, *p *= 0.646; control: *r *= 0.17, *p *= 0.194). Trait compulsivity was marginally associated with compulsive cocaine use (*r *= 0.34, *p *= 0.068), but not with eating behavior.

## Discussion

We provide evidence for a perturbation in fat regulation in cocaine-dependent men. Cocaine use was associated with reductions in body weight and these were remarkably specific to fat mass. It is clear that this effect reflects a more fundamental disturbance than can be accounted for simply by increased physical activity or reduced food intake, since lean mass did not differ across groups. We also found a trend towards lower levels of circulating leptin in the cocaine group, a protein hormone related to regulating energy expenditure and appetite. A decrease in plasma leptin together with a high fat diet suggests an impaired energy balance, which typically leads to weight gain rather than weight loss (Ainslie, Proietto, Fam, & Thorburn, 2000). The cocaine-dependent men in our study reported increased food intake, specifically in foods that are high in fat and carbohydrates, but there was no concomitant increase in body weight. In short, our findings challenge the widely held assumptions that cocaine use leads to weight loss through a global suppression of appetite. Rather, they suggest a profound metabolic alteration that needs to be taken into account if we are to understand fully the deleterious physical consequences of repeated use of this drug. A possible mechanism underlying the perturbation in fat regulation in the cocaine group might be related to the sympathetic effects of cocaine (Vongpatanasin, Mansour, Chavoshan, Arbique, & Victor, 1999) which may tonically inhibit leptin production (Rayner & Trayhurn, 2001) and facilitate overeating (Ainslie et al., 2000).

The specific reduction in body fat in the cocaine group is striking, given that the majority of the cocaine-dependent individuals have been long-term tobacco smokers, and chronic cigarette smoking has been associated with increased visceral fat in spite of relatively low body weight (Chiolero, Faeh, Paccaud, & Cornuz, 2008). This observation further strengthens our hypothesis that chronic cocaine use selectively reduces body fat deposition. We also investigated possible effects of the higher rate of past tobacco smoking in the control group by including smoking status as a covariate in the model. Cessation of tobacco smoking has been associated with weight gain (Filozof, Fernandez Pinilla, & Fernandez-Cruz, 2004), but we did not find evidence that the higher number of former smokers in the control group confounded the results.

Of course, the FFQ is a self-report measure and therefore susceptible to measurement error. While supplemental objective data such as nutritional biomarkers would have been desirable, we have no reason to assume that either control or cocaine-dependent volunteers would have been dishonest in their responses. Indeed, it is worth noting that, in prior studies, reports of personal information have repeatedly been shown to be reliable in non-treatment seeking drug users (Elman et al., 2000; Johnson et al., 2000). We therefore believe that the reported increase in food intake in the cocaine group is a real one and may reflect the relationship between the uncontrolled intake of fatty food and binge use of cocaine, which has previously been reported in experimental animals (Puhl, Cason, Wojnicki, Corwin, & Grigson, 2011). Hence cocaine-dependent individuals in the present sample report having problems in controlling their food intake, which may in part explain their high caloric intake. Their reduced effort of dietary restriction to lose weight paired with high levels of uncontrolled food intake confirms that cocaine-dependent men were not dieting, thus refuting the notion of cocaine being used as a means to suppress appetite and to lose weight (Cochrane et al., 1998). Overeating has been noted during the early stages of recovery in abstinent drug users, but not in active users, which has led to speculations that food might be used as a drug substitute (Cowan & Devine, 2008). Critically, however, the present data suggest that overeating in cocaine-dependence individuals pre-dates the recovery process, this effect being disguised by a lack of weight gain.

### Conclusion

Our data, in short, suggest that chronic cocaine abuse directly interferes with metabolic processes, resulting in an imbalance between fat intake and storage. This imbalance in active users could lead to excessive weight gain when the use of cocaine is discontinued during recovery. As the dysfunction in fat regulation is not detectable by conventional anthropometry measures, it is likely to be unnoticed in clinical practice. Given that the ability to control behavior is amenable to noradrenergic modulation (Chamberlain et al., 2006), changes in noradrenergic tone might offer a novel avenue for a therapeutic intervention, by altering the basal metabolic rate and ensuing energy expenditure as well as by improving inhibitory processes. Such intervention, at a sufficiently early stage, could have the potential to prevent weight gain during recovery, thereby reducing personal suffering and improving compliance during the recovery process. This is an important consideration given that by far the most substantial health burden arising from drug addiction lies not in the direct effects of drug intoxication but in the secondary effects on physical health.

## Financial disclosures

This study was funded by a research Grant from the Medical Research Council (G0701497) and conducted within the Behavioural and Clinical Neuroscience Institute (jointly funded by the Medical Research Council and the Wellcome Trust). K. D. Ersche is supported by the Medical Research Council. The software used for the processing of the Food Frequency Questionnaire data was developed by EPIC-Norfolk study, which is supported by programme Grants from the Medical Research Council UK and Cancer Research UK.

## Figures and Tables

**Table 1:** Group differences with regard to clinical characteristics and anthropometry.

	Healthy men (*N* = 30)		Cocaine-dependent men (*N* = 35)		Group comparison	
Mean	Std.	Mean	Std.	*t* or U	Sig.
Plasma leptin (μg/L)	4.7	±5.4	2.9	±3.0	-1.7	0.093
Systolic blood pressure (mm Hg)	131.7	±15.7	131.2	±16.5	0.1	0.895
Diastolic blood pressure (mm Hg)	79.1	±9.6	75.0	±13.0	0.4	0.154
Pulse rate (per minute)	70.3	±13.5	67.9	±12.0	0.8	0.443
Waist girth (Inch)	89.4	±10.7	84.7	±8.8	3.0	0.089
Hip girth (Inch)	98.8	±7.3	95.7	±7.1	2.3	0.131
Waist–hip-ratio (WHR)	0.90	±0.07	0.89	±0.06	1.0	0.319
Triceps skin-fold thickness (mm)	11.4	±4.4	9.4	±4.2	2.6	0.110
**Biceps skin-fold thickness** (mm)	**7.1**	±**3.9**	**5.2**	±**2.0**	**5.4**	**0.023**
Subscapular skin-fold thickness (mm)	14.5	±5.5	11.7	±5.1	3.4	0.072
**Weight** (kg)	**80.1**	±**13.4**	**74.0**	±**10.7**	**2.0**	**0.046**
Height (m)	1.77	±0.08	1.76	±0.07	0.7	0.504
Body mass index (BMI)	25.4	±3.5	23.9	±3.4	1.8	0.081
**Fat** (%)	**24.8**	±**8.0**	**19.8**	±**8.7**	**2.4**	**0.021**
**Fat** (kg)	**20.3**	±**9.3**	**15.1**	±**8.3**	**2.4**	**0.020**
Fat-free mass (kg)	58.8	±7.4	57.6	±5.1	0.7	0.463
Lean (kg)	55.6	±7.0	54.6	±4.8	0.7	0.503
Bone mineral density (BMD, total)	3.2	±0.5	3.0	±0.4	1.5	0.151
